# Understanding desistance from aggression: A joint interpretation of person-centered and variable-centered approaches

**DOI:** 10.1017/S0954579425100382

**Published:** 2025-08-11

**Authors:** Sarah L. Carroll, Alaina M. Di Dio, Shaunna L. Clark, Kelly L. Klump, Luke W. Hyde, S. Alexandra Burt

**Affiliations:** 1 Department of Psychology & Neuroscience, University of Colorado Boulder, Boulder, CO, USA; 2 Department of Psychiatry, Texas A&M University, College Station, TX, USA; 3 Department of Psychology, Michigan State University, East Lansing, MI, USA; 4 Department of Psychology, Survey Research Center at the Institute for Social Research, University of Michigan, Ann Arbor, MI, USA

**Keywords:** aggression, desistance, developmental trajectories, person-centered, variable-centered

## Abstract

When leveraged together, variable-centered and person-centered statistical methods have the potential to illuminate the factors predicting mental health recovery. However, because extant studies have largely relied on only one of these methods, we do not yet understand why some youth demonstrate recovery while others experience chronic symptoms. This omission limits our understanding of trajectories of physical aggression (AGG) in particular, which are frequently characterized by desistance. The present study examined the development of AGG across childhood and adolescence via variable-centered and person-centered modeling, with neighborhood and family characteristics considered as predictors. Variable-centered results indicated a mean-level decline in AGG with age but were more useful for illuminating predictors of AGG at baseline than predictors of declining engagement. Person-centered analyses, by contrast, identified low parent-child conflict and high household income as predictors of desistance. Although variable-centered analyses were integral to modeling the average AGG trajectory and identifying predictors of engagement at baseline, person-centered techniques proved more useful for understanding predictors of desistance.

## Introduction

Physical aggression (AGG) encompasses a broad spectrum of behaviors that violate the personal rights of others, ranging from relatively minor acts (e.g., hitting, kicking) to more serious violent crimes (e.g., stabbing, shooting) that bring offenders into contact with the criminal justice system. In light of the personal and societal consequences associated with persistent AGG (Odgers et al., 2008), a large body of longitudinal work has sought to illuminate its development across childhood and adolescence. Such studies have yielded numerous conclusions about typical trajectories, perhaps most notably that AGG is so prevalent as to be normative during the preschool years, but that by late adolescence, engagement is limited to a small proportion of youth who exhibit poor adjustment across domains (e.g., Mazza et al., 2017; Tung & Lee, 2017). Such findings raise a key question for developmental researchers that has yet to be answered, namely, *why* do the majority of youth desist from AGG? That is, even though youth AGG has been studied extensively, the defining characteristic of desistance from AGG has not. As such, we know very little about the factors distinguishing youth with high levels of AGG who eventually desist from their counterparts whose engagement is persistent and increasingly severe.

**Figure 1.**
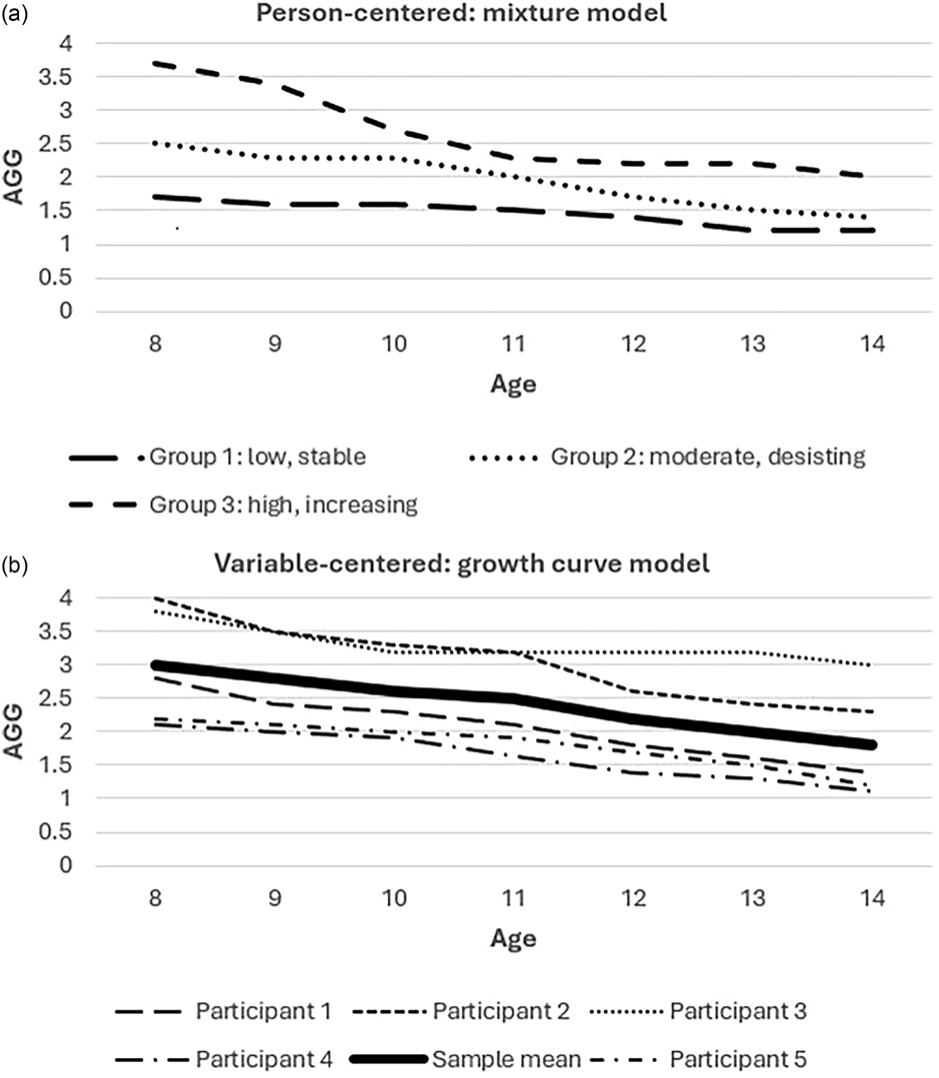
Person-centered (a) and variable-centered (b) approaches to modeling the development of AGG in a hypothetical childhood sample. The person-centered approach extracted three distinct trajectory groups, whereas the variable-centered analysis identified a mean-level trend across the entire sample.

Efforts to distinguish between desistance and persistence are well-suited for person-centered methodologies. These methods consider the sample to comprise distinct subgroups that differ by intercept and/or slope and thus allow us to identify discrete trajectory groups based on their patterns of change over time. Most of these studies have relied on a technique developed by Nagin (1999), termed semi-parametric mixture modeling or latent class growth analysis (LCGA). In this approach, participants are assigned to latent, or unobserved, trajectory groups based on their posterior probability of group membership (i.e., their likelihood of belonging to a particular group given their scores on the variable of interest) (see Figure 1a for hypothetical results from a person-centered model). Patterns of continuity and change are captured by the intercept, or level of the outcome variable at a particular point in time (often at baseline), and the slope, or rate of change. For example, a participant who reports escalating levels of AGG would likely be assigned to a trajectory group with a positive slope, whereas a participant who reports frequent engagement only at baseline would likely be assigned to a desisting trajectory, with a negative slope. Although groups derived via LCGA are free to vary from one another by intercept and slope, in most studies, participants within the same group are assumed to follow the same trajectory (i.e., intercept and slope variances are fixed within groups).

The results of these kinds of analyses have been consistent across the literature, with most studies identifying three or four trajectories of AGG and a modal group that follows a declining trajectory by middle childhood (Carroll et al., 2023). As an example, Côté and colleagues (2006) identified three AGG trajectories in a nationally representative sample of more than 10,000 Canadian children. Most youth were assigned to the moderate/desisting group, which followed a trajectory of declining AGG across the study period (ages two to 11 years). Less than one-third of the sample exhibited consistently low AGG, and the remaining 16% followed a trajectory of chronic engagement, consistent with the broader literature indicating that desistance, rather than either persistence or complete abstention, is the norm. Extant person-centered literature also sheds light on predictors of AGG trajectories (although not on predictors of desistance from AGG per se). In the study by Côté et al., for example, male sex, familial socioeconomic disadvantage, and hostile parenting predicted membership in the chronic trajectory relative to the two lower-risk groups together (the study did not examine predictors of membership in the chronic trajectory relative to the desisting trajectory by itself). Indeed, most person-centered studies examining sex differences have reported males to be overrepresented in high-risk trajectories (e.g., Wildeboer et al., 2015). Exposure to disadvantage in the broader neighborhood context (e.g., Census tract poverty, community violence) has also been linked to trajectories of persistent (Spano et al., 2010) and increasing (Lacourse et al., 2008) AGG.

However, person-centered methods such as LCGA represent only one of two primary statistical approaches that have been leveraged in longitudinal studies of AGG. The other, “variable-centered” approach explicitly conceptualizes the sample as a single population in which participants vary continuously from one another in their trajectories. This method employs growth curve modeling to quantify the mean rate of change over time across the sample (see Figure 1b for hypothetical results from a variable-centered model) (McArdle & Epstein, 1987). In other words, the means of the intercept and slope represent the average pattern of development across the full sample, whereas the variances represent individual differences in the initial level of the outcome and rate of change, respectively (Preacher et al., 2008). For example, a variable-centered analysis of AGG could find the mean linear slope to be negative and the intercept and slope variances to each be significant. These findings would indicate that the average participant exhibited a steady decline in AGG with age, but that there were significant between-person differences in both initial level of AGG and rate of change.

Indeed, extant variable-centered studies have generally reported this pattern during middle childhood and adolescence. That is, although mean-level increases in AGG are normative during the preschool years, desistance becomes the norm as children progress through school. As one representative example, Lee and colleagues (2016) modeled the development of AGG in a community-based sample via growth curve modeling and found that the mean level of AGG decreased from ages seven to 19 years, albeit with significant variation between youth in their baseline engagement and rate of change. These results dovetail nicely with the conclusions from the person-centered literature, particularly regarding the normative nature of desistance.

Variable-centered studies of the predictors of AGG have also yielded results that complement those from the person-centered literature. For instance, male sex predicted greater AGG at baseline in an adolescent sample from Chicago (Sacco et al., 2015), consistent with reports both from person-centered studies and from the broader variable-centered literature indicating significant sex differences in the development of AGG (Carroll et al., 2023). Sacco and colleagues found that lower neighborhood income also predicted higher baseline AGG (Sacco et al., 2015). Indeed, both neighborhood and familial socioeconomic deprivation are consistently observed to predict the intercept in growth curve models, in the expected direction (Carroll et al., 2023; Olson et al., 2018), as are other aspects of the family environment (e.g., harsh discipline (Olson et al., 2018) parent-child hostility (Benson & Buehler, 2012)). Notably, however, most variable-centered studies of AGG have reported greater differences in the intercept than in the slope as a function of the moderators under study (e.g., sex, neighborhood disadvantage (Carroll et al., 2023)), despite the high likelihood that youth who desist differ from those exhibiting persistent AGG on numerous individual and contextual characteristics.

As can be gleaned from the studies reviewed above, neither variable-centered nor person-centered research has yet to substantially advance our understanding of desistance. This gap may in part reflect the fact that each model provides only a *partial* picture of the development of AGG (Carroll et al., 2023). The growth curve representing average change in AGG in variable-centered studies, for example, may not adequately capture the developmental heterogeneity observed in key participants. That is, even if the mean slope indicates a modest decline over time, some youth might persist in their delinquency over time while others may fully desist. In other words, there can be more information in the slope variance than in the slope mean. The primary strength of person-centered methods lies in their ability to illuminate heterogeneity in the slope variance, identifying specific groups of youth whose patterns of engagement make them important targets for intervention efforts. As such, these methods have a clear potential to identify the factors that distinguish desisting trajectories from persistent ones, though very few studies to date have done so. Nevertheless, person-centered approaches can be limited in their ability to identify predictors of baseline AGG, particularly when the trajectory groups have similar intercepts. Although not central to the study of desistance per se, identifying predictors of the intercept is a key part of understanding trajectories of AGG, given that the children with the greatest engagement at baseline will likely exhibit higher levels than their peers throughout early development (Burt, 2012). Person-centered methods are also limited by the fact that most forms of psychopathology, including AGG, appear to be dimensional in nature across the population, highlighting a mismatch between these modeling approaches and the way in which AGG is actually distributed. Instead, AGG is likely modeled more accurately using variable-centered methods, which consider all participants to be drawn from the same underlying population (Walters & Ruscio, 2013; Walters, 2011). In short, we contend that both variable-centered and person-centered approaches are needed to model the development of AGG in a way that matches the underlying distribution of the trait *and* illuminates the key predictors of desistance.

A recent review (Carroll et al., 2023) began the process of jointly interpreting variable-centered and person-centered findings through a conceptual review of the developmental literatures on antisocial behavior. Of the 124 studies of AGG included in the review, only four (3%) leveraged variable and person-centered techniques simultaneously (Dong, 2015; Ehrenreich et al., 2014; Ingoldsby, 2002; NICHD Early Child Care Research Network, 2004). The results of these studies yielded little information about the predictors of desistance from AGG. Two did not examine predictors of the slope variance (Dong, 2015; Ehrenreich et al., 2014), and one was under-powered (Ingoldsby, 2002; *N* = 170). Only one study identified specific predictors of desistance, which included higher maternal educational attainment and fewer maternal depressive symptoms, but participants were not followed past age nine (NICHD & Arsenio, 2004). Indeed, no study to date has employed both person-centered and variable-centered techniques to examine predictors of desistance from AGG from childhood through to early adulthood. Such studies would be crucial for identifying targets to leverage in intervention efforts for youth demonstrating elevated levels of AGG, who comprise a sizeable proportion (∼10% to >50%; e.g., Broidy et al., 2003; Nagin et al., 2009) of childhood samples.

We note that the criminology literature, in contrast to the literature on youth AGG, has often focused on the factors predicting desistance relative to recidivism. For example, transitioning into adult roles, such as marriage, has consistently been implicated as a predictor of desistance from violent crime (Walker et al., 2013). Nevertheless, there are several characteristics of the criminology literature that limit the conclusions we can draw about desistance from AGG. First, studies typically examine crime as the outcome measure in samples of adjudicated youth. These samples are not representative of the general population but rather capture behavioral trajectories that are severe enough to result in legal consequences. The legal consequences themselves are most often the outcome under study, in contrast to the measures used in studies of AGG, which typically assess the presence or frequency of aggressive behaviors. Assessment of the consequences, rather than the presence, of behavioral problems has several limitations for our understanding of behavioral trajectories (see Burt, 2022 for a detailed discussion). For example, there is robust and consistent evidence of disparities in policing and adjudication across ethnic/racial identities (e.g., Pierson et al., 2020). Item response theory work has also identified disparities in questionnaire data, such that history of arrest was highly predictive of one’s standing on a latent delinquency trait for White adolescents, but not for Black adolescents, in a sample of over 7,500 youth (Brislin et al., 2024). In addition, studies examining legal consequences necessarily omit less severe behaviors, meaning they do not capture the full dimension of AGG. Rather, the measures used in criminology studies align with a categorical conceptualization of antisocial behavior, despite the consistent finding that data on youth antisocial behavior are distributed dimensionally (Walters & Ruscio, 2013; Walters, 2011). Consistent with their focus on severe behaviors, criminology studies can identify youth who desist from crime, but this does not necessarily indicate desistance from AGG. Indeed, even those youth who cease to commit crimes often experience poor behavioral health outcomes (Moffitt et al., 2002; Sampson & Laub, 2003).

Second, although clearly related, AGG and violent crime are quite different phenotypes, given their differing severities. Indeed, in their review of the literature on desistance from violence, Walker et al. (2013) note the likely existence of two age-crime curves, one representing AGG and the other representing violent crime. The former, which encompasses acts that would likely not be considered crimes (e.g., hitting, kicking), is suggested to peak in early-to-middle childhood, consistent with the literature reviewed above. The latter, by contrast, peaks in late adolescence, when engagement in crime is both most frequent and most prevalent (Hirschi & Gottfredson, 1983). Notably, the age-crime curve refers to the *frequency* of engagement in crime (or AGG), not the severity. Severity, which is considered much less often than frequency in studies of antisocial behavior, typically refers to the impact of the behavior on the victim (Burt, 2022). Although the frequency of AGG begins to decline by the late preschool years, severity increases with age, facilitated by age-related increases in strength and ability to use weapons (Burt, 2012). Criminological studies necessarily focus on the adolescent-peaking age-crime curve, to the exclusion of physically aggressive acts of lesser severity that onset early in life. Such acts are likely precursors to engagement in violent crime, and those youth who fail to desist from AGG are likely the same youth who go on to commit crimes. Studies leveraging dimensional measures of AGG are needed to identify not only the small proportion of youth who exhibit persisting AGG (that is likely increasingly severe), but also the substantial proportion who engage in some degree of AGG during childhood, whose behaviors would be overlooked in studies assessing crime.

Despite the differences between AGG and violent crime, theoretical considerations from criminology prove useful for envisioning what desistance may entail. One question raised in the literature is whether the factors predicting desistance are merely the opposite of those predicting onset. In their chapter on desistance from crime, Sampson & Laub (2003) proposed two distinct schools of thought, one in which the “reverse” of risk factors indeed predicted desistance and another in which onset and desistance were related to entirely different factors. In the latter, they suggested that, for example, peer influences may contribute to onset of criminal activity whereas entry into adult social and occupational roles may effect desistance. For AGG, the extent of overlap in the factors predicting baseline engagement and desistance, respectively, is unknown. In light of the utility of variable-centered approaches for identifying predictors of baseline engagement and person-centered approaches for illuminating the slope variance, we believe that leveraging both approaches provides an optimal framework for evaluating these competing hypotheses.

### Present study

The present study addressed these gaps in the literature by leveraging both variable-centered and person-centered approaches to illuminate the development of AGG from childhood through to early adulthood, with a focus on identifying the predictors of desistance. Participants were drawn from a large, population-based accelerated longitudinal sample enriched for exposure to neighborhood disadvantage. The development of AGG was modeled via a series of variable-centered and person-centered analyses, and biological sex, household income, parenting, and neighborhood disadvantage were examined as predictors of AGG growth curves and trajectories. We examined socioeconomic disadvantage at both the family and neighborhood levels, in light of prior research indicating that each form of disadvantage has unique implications for youth behavioral outcomes, particularly externalizing (Carroll et al., 2024). Consistent with prior work (e.g., Brame et al., 2001; Lee et al., 2016), we hypothesized that 1) the mean level of AGG would decline across the study period, 2) there would be either three or four AGG trajectory groups, and 3) female sex, higher household income, greater parental nurturance, lower parent-child conflict, and greater neighborhood advantage would predict lower levels of AGG at baseline as well as desistance from AGG across adolescence.

### Transparency and openness

The present study was not preregistered. Because of the language in the informed consent document at intake, we cannot post the data publicly, but they can be obtained from the primary author upon reasonable request. We report how we determined our sample size, all data exclusions, all manipulations, and all measures in the study. All procedures were approved by Michigan State University’s institutional review board and are in compliance with the ethical standards of the relevant national and institutional committees on human experimentation and with the Helsinki Declaration. Child participants provided informed assent, and parents provided informed consent for themselves and their children.

## Methods

### Participants

The population-based Michigan State University Twin Registry (MSUTR) includes several independent twin projects. Participants in the current study were drawn from the Twin Study of Behavioral and Emotional Development in Children (TBED-C), a study within the MSUTR. The TBED-C includes a population-based arm (*N* = 528 families), and an “under-resourced” arm for which inclusion criteria also specified that participating twin families lived in neighborhoods with neighborhood poverty levels at or above the Census mean at study onset (10.5%) (*N* = 502 families) (total *N* = 1,030 families and 2,060 children, as both twins always participated together). To recruit families at intake, the Department of Vital Records in the Michigan Department of Health and Human Services (MDHHS) identified twins in our age range via the Michigan Twins Project (MTP), a population-based registry of more than 32,000 twins in Lower Michigan recruited via birth records. The Michigan Bureau of Integration, Information, and Planning Services database was used to locate family addresses no more than 120 miles from East Lansing, Michigan through parent drivers’ license information. Pre-made recruitment packets were then mailed to parents by the MDHHS. Parents who did not respond to the first mailing were sent additional mailings roughly one month apart until either a reply was received or up to four letters had been mailed.

This recruitment strategy for the TBED-C yielded an overall response rate of 57% for the at-risk sample and 63% for the population-based sample. Other recruitment and sampling details can be found in prior publications (i.e., Burt & Klump, 2019). The two arms of the study were analyzed jointly for the current analyses and were 48.7% female and 51.3% male. Across the full sample, participants endorsed racial/ethnic identities in the following proportions: white (non-Latinx), 82%; Black, 10%; Latinx, 1%; Asian, 1%; Indigenous, 1%; multiracial, 6%. However, families in the under-resourced arm, but not the population-based arm, were more racially diverse than the local population (e.g., 14% Black and 77% white in the under-resourced arm versus 5% Black and 87% white in the population-based arm; 5% Black and 85% white in the local area Census).

Because 90+% of TBED-C families were recruited out of the MTP, we were able to use the MTP data to compare families who chose to participate in TBED-C with those who were recruited but did not participate. TBED-C families were generally representative of recruited but non-participating families. As compared to non-participating twins, participating twins reported similar levels of conduct problems, emotional symptoms, and hyperactivity (*d* ranged from −.08 to .01 in the population-based arm and .01 to .09 in the under-resourced arm; all ns). Participating families also did not differ from non-participating families in paternal felony convictions (*d* = −.01 and .13 for the population-based and under-resourced arms, respectively), rate of single parent homes (*d* = .10 and −.01 for the population-based and under-resourced arms, respectively), paternal years of education (both *d* ≤ .12), or maternal and paternal alcohol problems (*d* ranged from .03 to .05 across the two arms). However, participating mothers in both samples reported slightly more years of education (*d* = .17 and .26, both *p* < .05) than non-participating mothers. Maternal felony convictions differed across participating and non-participating families in the population-based arm (*d* = −.20; *p* < .05) but not in the under-resourced arm (*d* = .02). In short, our recruitment procedures appear to have yielded a sample that was representative of both recruited families and the general population of the State of Michigan.

Behavioral data regarding the children’s AGG were collected at up to three time points. All 1,030 families were assessed once in middle childhood (ages 5 – 12; M_age_ (SD) = 8.23 (1.52)) as part of the TBED-C (Wave 1). TBED-C participants residing in modestly-to-severely disadvantaged neighborhoods (*N* = 768 families) are currently being recruited for reassessment as adolescents up to two times, 18-months apart, through the Michigan Twin Neurogenetics Study (MTwiNS). Thus, all families from the under-resourced arm of the TBED-C are being recruited for participation in MTwiNS, as are those from the population-based arm who reside in neighborhoods with poverty rates at or above 10.5%. As a result, 55% of the sample has participated in only one assessment wave. The first of the MTwiNS assessments was conducted approximately 4 − 6 years after participation in TBED-C (ages 7 − 24; M_age_ (SD) = 15.43 (2.62)), while the second adolescent assessment was 5 − 7 years after participation in TBED-C (ages 10 – 23; M_age_ (SD) = 16.50 (2.42)). For the present study, the sample sizes at Waves 2 and 3 were 950 and 604, respectively. The TBED-C and MTwiNS studies had an accelerated longitudinal design, with age ranges that largely overlapped across assessment waves (i.e., ages 5 − 12, 7 − 24, and 10 − 23 years at Waves 1, 2, and 3, respectively). In light of this, we examined the development of AGG as a function of age, rather than assessment wave, in all longitudinal analyses here.

### Procedure

TBED-C families completed their intake assessments between 2008 and 2015. Assessment teams consisted of two research assistants and at least one paid staff member and took 4 – 5 hours to complete (lunch was provided). Families completed questionnaires, interviews, and videotaped interactions during the in-person assessment. The primary caregiver (nearly always the mother) participated alongside the twins. Assessments typically took place in the East Lansing-based university laboratory (903 families). If families were unable or unwilling to travel to the university, assessments took place in participants’ homes (127 families; families with younger twins were more likely to complete home visits, as were families that identified as white). The MTwiNS follow-up assessments were also completed in-person. The twins and their primary caregiver completed an in-person assessment lasting 4 − 8 hours at either the Ann Arbor or East Lansing-based laboratories (most were completed in Ann Arbor). The twins completed a series of structural and functional neuroimaging tasks, as well as a battery of child-report questionnaires. Caregivers, in turn, participated in an interview and completed a series of questionnaires. Additional details are reported in prior publications (e.g., Burt & Klump, 2019; Tomlinson et al., 2022).

### Measures

#### AGG

Youth AGG was defined via parent report. The primary caregiver (nearly always the twins’ mother) completed the 18-item AGG scale from the Child Behavior Checklist (CBCL; Achenbach & Rescorla, 2001) at all three waves, rating the extent to which a series of statements described the child’s behavior over the past six months using a three-point scale (0 = never to 2 = often/mostly true). Caregiver-informant reports were available for 99% of participating twins at Wave 1, 96% at Wave 2, and 97% at Wave 3. One issue with the AGG scale from the Achenbach family of instruments is that the items assess not only physically aggressive behaviors (e.g., fights, destroys belongings) but also verbal AGG (e.g., argues) and emotional dysregulation (e.g., sudden changes in mood). Because the aim of our study was to model the development of physical AGG, we constructed a shortened scale of items from the Achenbach instruments via a series of item response theory (IRT) analyses. We retained eight items from the CBCL that were face-valid and assessed a range of physically aggressive behaviors (e.g., gets in many fights, hot temper) (see Supplementary Methods for more details).

#### Familial context

Familial socioeconomic deprivation was assessed via maternal reports of annual household income at the TBED-C assessment. Household income was measured on a 10-point Likert scale (1 = <$10,000 to 10 = >$50,000).

At the TBED-C assessment, participating mothers and children also completed the Parental Environment Questionnaire (PEQ; Elkins et al., 1997), which assesses several dimensions of the parent-child relationship using a 4-point Likert scale ranging from “definitely false” to “definitely true.” We focused on the Conflict and Nurturance scales (12 items each; *α* ≥ .68 for all scales and informants). The Conflict scale assesses harsh or conflictive parenting practices (e.g., “I often criticize my child”), while the Nurturance or Involvement scale assesses parental communication, support, and involvement with their child (e.g., “I praise my child when he/she does something well”). Items are nearly identical across parent and child versions of the PEQ, with minor alterations in wording. Responses were coded so that higher scores indicated higher levels of each construct. Scores were averaged across informants to create composite reports of conflict and nurturance, respectively. Prior work has found the Conflict and Nurturance scales from the PEQ to demonstrate strong internal consistency reliability for parents and children and to be highly correlated with scores on the Family Environment Scale, which also assesses quality of the parent-child relationship (Elkins et al., 1997).

#### Neighborhood context

Neighborhood disadvantage was defined via the Area Deprivation Index (ADI; Kind & Buckingham, 2018), which comprises 17 indices of Census block group disadvantage (e.g., poverty rate, income disparity; see Supplementary Table 1). Data were weighted according to the factor loadings identified by Kind & Buckingham (2018), and weighted variables were summed to create a deprivation index score for each Census block group. Families were assigned a percentile indicating the level of deprivation in their block group relative to that of all U.S. block groups. Mean ADI scores were 57.24 (SD = 22.67; range: 2 – 99), 61.57 (SD = 20.94; range: 6 – 99), and 61.25 (SD = 21.68; range: 4 – 100) at the TBED-C and MTwiNS assessments, respectively. These scores correspond to a moderate level of neighborhood disadvantage, albeit with considerable variation across participating families. At the TBED-C assessment, for instance, Census tract poverty rates ranged from 0 to 93%, with a mean of 19%.

#### Race/Ethnicity

Given inequalities in exposure to socioeconomic disadvantage across racial/ethnic identities (Peterson & Krivo, 2009), race was included as a covariate in all longitudinal analyses, coded dichotomously as white/racially marginalized due to the composition of our sample.

### Data analytic strategy

Sum scores for parent-reported AGG were computed based on the items retained in the final IRT model (see Supplementary Methods and Tables S3 – S6 for additional details). These scores were log-transformed to adjust for positive skew and were subsequently examined as the outcome measures in the variable-centered and person-centered analyses. Continuous covariates (i.e., income, parenting, and neighborhood disadvantage) were standardized for ease of interpretation. All analyses accounted for the clustering of twins within families using the CLUSTER command. There was relatively little clustering, however, at the neighborhood level; among the 1,030 participating families, more than 700 Census tracts were represented. As a result, we did not model clustering of families within neighborhoods. In all longitudinal analyses, age was examined in lieu of assessment wave as the metric of time.

#### Variable-centered analyses

Analyses were conducted in M*plus* 8.4 (Muthén & Muthén, 1998 –2019) using a robust maximum likelihood estimator and full information maximum likelihood estimation (FIML) to account for missing data on the outcome, as prior simulations have shown FIML to be robust to at least 50% missing data (Enders & Bandalos, 2001). In addition, FIML is recommended as a gold standard for handling missing data in accelerated longitudinal designs (Little & Rhemtulla, 2013). We examined phenotypic changes in the frequency of AGG over time across the full sample via latent growth curve modeling, which identifies within-person changes in the outcome of interest as well as between-person variation in these changes (McArdle & Epstein, 1987). First, unconditional models were run to determine whether engagement in AGG increased, decreased, or remained constant with age. We made use of the TSCORES option in M*plus* to model age as the metric of time as it allows for individually-varying times of observation (see M*plus* User’s Guide for more details).

Intercepts were centered to represent AGG at age 5.6, the youngest age at the first (TBED-C) assessment wave. The slope factor represented annual change. Because participants were assessed on only three occasions, we could not examine non-linear growth. Initial analyses compared the respective fits of an intercept-only (“no-growth”) model and a model allowing for linear growth. The former had three parameters (intercept mean, intercept variance, and residual variance), and the latter had six (intercept and slope means, intercept and slope variances and their covariance, and residual variance). Model fit was evaluated with four likelihood-based indices: the chi-square difference test (Bollen, 1989), Akaike information criterion (AIC; Akaike, 1987), Bayesian information criterion (BIC; Raftery, 1995), and sample-size adjusted Bayesian information criterion (SABIC; Sclove, 1987). For all indices, lower values indicated better model fit.

We selected the best-fitting model for the conditional growth curve analyses. AGG was fit conditional upon sex, race, income, conflict, nurturance, and ADI scores, all of which were examined as time-invariant predictors. Because M*plus* employs listwise deletion for missing covariates, the means and variances of all covariates were mentioned in the model so that they would be treated as dependent variables for the purposes of missing data. As a result, missing data for the covariates were accounted for using FIML rather than listwise deletion, due to FIML’s documented robustness with large amounts of missing data (Enders & Bandalos, 2001).

#### Person-centered analyses

Latent class growth analyses were conducted in M*plus* 8.4 using a robust maximum likelihood estimator and FIML to account for missing outcome data. We examined participants’ growth in AGG as a function of their age at each assessment wave using the TSCORES option. We began with a one-class model and progressed through to a six-class model, consistent with the steps outlined by Muthén (2004) and with the approach employed in prior studies examining trajectories of antisocial behavior (e.g., Odgers et al., 2008). Consistent with the dominant model in past studies (Nagin, 1999), all models allowed the groups to differ from one another by intercept and slope but constrained within-group intercept and slope variances to zero. The optimal model was determined via consideration of three fit indices, the AIC, BIC, and SABIC, as well as the interpretability of the class solution (i.e., the percentage of the sample assigned to each class). Biological sex, race, income, parenting, and ADI were subsequently examined as time-invariant predictors of class membership using the M*plus* R3STEP command, which accounts for classification uncertainty (Asparouhov & Muthén, 2013).

#### Missing data analyses

In light of the study’s accelerated longitudinal design with planned missingness, we ran a series of analyses to check for differential attrition. Analyses were conducted in SPSS 28.0. We made use of multilevel modeling (MLM), with a random intercept to account for the nesting of twins within families, to examine differences in Wave 1 reports of AGG, conflict, nurturance, and sex as a function of the number of assessments completed. Because twins within a pair were necessarily concordant for household income and neighborhood deprivation, we examined differences in these variables as a function of assessments completed through a series of one-way analyses of variance (ANOVAs), with the family as the unit of analysis.

In these analyses, the number of assessments completed did not predict participants’ Wave 1 AGG (*F*(2, 1050.405) = .694, *p* = .500) or parent-child conflict (*F*(2, 1027.954) = .629, *p* = .533). However, nurturance did differ significantly as a function of number of assessments completed (*F*(2, 1025.578 = 7.893, *p* < .001). Pairwise comparisons indicated that participants with only one wave of data reported higher nurturance compared to those with three waves of data (mean difference = .855, *SE* = .218, *p* < .001). There were also sex differences in the number of assessments completed (*F*(2, 2040) = 3.94, *p* = .020), such that those with two waves of data were more likely to be male relative to those with either one wave (estimate = .099, *SE* = .045, *p* = .027) or three waves (estimate = .142, *SE* = .050, *p* = .005).

We also observed expected differences in neighborhood disadvantage (*F*(2, 998) = 20.889, *p* < .001, eta-squared = .040) based on the number of assessments completed. Specifically, those who participated in only one wave had lower ADI scores, indicating less neighborhood deprivation, on average than did those participating in two or three waves (mean differences = −7.947 and −9.770, respectively, *ps* < .001). This difference was expected given that the two adolescent assessments were restricted to families residing in under-resourced neighborhoods. Household income did not differ as a function of the number of assessments completed (*F*(2, 998) = 1.415, *p* = .243, eta-squared = .003). Altogether, the differences between participants as a function of attrition were small in magnitude (eta-squared values ranged from .003 – .04). In other words, despite the accelerated longitudinal design with planned missingness, we expect that the effects of differential attrition on study results would be minimal.

## Results

### Descriptives

Descriptive statistics and correlations for the AGG sum scores are shown in Table 1. Participants evidenced moderate rank-order stability in AGG over time. Moreover, paired-sample *t* tests indicated that AGG decreased in frequency from Wave 1 to Wave 2 (*t*(898) = 16.31, *p* < .001), but not from Wave 2 to Wave 3 (*t*(574) = 1.30, *p* = .195). At Waves 1 and 2, males had higher levels of AGG than females (Cohen’s *d* = .285, *p* < .001 and Cohen’s *d* = .135, *p* = .042, respectively); although in the expected direction, sex differences were not significant at Wave 3. In addition, ADI and income were significantly correlated with AGG at the first two time points, but not at the third. Conflict and nurturance were significantly correlated with AGG at all waves. All correlations were in the expected direction. (See Table S2 for descriptives by age, rather than wave).

**Table 1.** Descriptive statistics and correlations

	1.	2.	3.	4.	5.	6.	7.
ADI	–						
Income	**−.40**	–					
Conflict^a^	.02	−.01	–				
Nurturance^a^	**−.15**	**.13**	**−.37**	–			
AGG T1^b^	**.15**	**−.16**	**.35**	**−.16**	–		
AGG T2^b^	**.07**	**−.11**	**.18**	**−.09**	**.36**	–	
AGG T3^b^	.02	−.06	**.18**	**−.09**	**.35**	**.58**	–
Mean (SD)	57.25 (22.68)	8.24 (2.68)	20.66 (4.63)	41.45 (3.53)	1.58 (1.88)	.62 (1.18)	.59 (1.28)
Range	2 – 99	1 – 10	12 – 39	22 – 48	0 – 8	0 – 8	0 – 8
*N*	2010	2010	2041	2041	2048	913	585

Bold font indicates *p* < .05. ^a^Mean scores were computed based on maternal and child reports. ^b^Sum scores were computed based on maternal reports on eight CBCL items.

### Variable-centered

#### Unconditional models

Initial growth curve analyses tested the hypothesis that the frequency of AGG decreased with age by comparing the respective fits of an intercept-only model and a linear growth model. The linear growth model fit better than the intercept-only model according to all fit indices (see Table 2) and was thus selected as the final unconditional model. At age 5.62, the average participant had a score of .830 on AGG (SE = .022, *p* < .001). On average, scores decreased by .050 points per year (SE = .002, *p* < .001), consistent with hypotheses. Variation between persons was significant for baseline level (*b* = .191, SE = .043, *p* < .001) but not for rate of change (*b* = .000, SE = .000, *p* = .326). The estimated covariance between the intercept and slope was nonsignificant (*b* = −.006, SE = .004, *p* = .160).

**Table 2.** LGM fit statistics and parameter estimates

	Unconditional LGM model fit statistics					
	Model	−2lnL	*χ* ^*2*^ (df)	AIC	BIC	SABIC
	**Linear growth**	**6041.81**	**-**	**6057.81**	**6102.82**	**6077.41**
	Means model	6575.78	533.97† (3)	6585.78	6613.91	6598.03
	**Conditional LGM parameter estimates**					
	Parameter	Estimate	S.E.	*p*-value		
**Intercept**	**Mean**	**.726**	**.058**	**< .001**		
	**Variance**	**.168**	**.037**	**< .001**		
	**ADI**	**.057**	**.023**	**.013**		
	**Income**	**−.070**	**.025**	**.006**		
	**Conflict**	**.299**	**.020**	**< .001**		
	Nurturance	.038	.020	.052		
	**Sex**	**.189**	**.039**	**< .001**		
	Race	.009	.060	.886		
**Slope**	**Mean**	**−.044**	**.007**	**< .001**		
	Variance	.001	.000	.092		
	ADI	−.004	.003	.147		
	Income	.002	.003	.468		
	**Conflict**	**−.019**	**.002**	**< .001**		
	Nurturance	−.003	.002	.236		
	**Sex**	**−.017**	**.005**	**< .001**		
	Race	.002	.007	.750		
	Intercept with slope	−.007	.004	.072		
	**Wave 1 Residual Variance**	**.244**	**.025**	**< .001**		
	**Wave 2 Residual Variance**	**.131**	**.012**	**< .001**		
	**Wave 3 Residual Variance**	**.123**	**.023**	**< .001**		

Bold font indicates *p* < .05. †Significant change in chi-square at *p* < .05.

#### Conditional model

Sex, race, income, conflict, nurturance, and ADI were subsequently examined as predictors of continuity and change in AGG. As hypothesized, male sex, lower income, greater parent-child conflict, and higher ADI predicted greater AGG at baseline (see Table 2). Race and nurturance predicted neither baseline AGG nor rate of change. The only significant predictors of the slope were sex and parent-child conflict, with males and those with greater conflict demonstrating a more rapid age-related decline.

### Person-centered

#### Unconditional models

**Figure 2.**
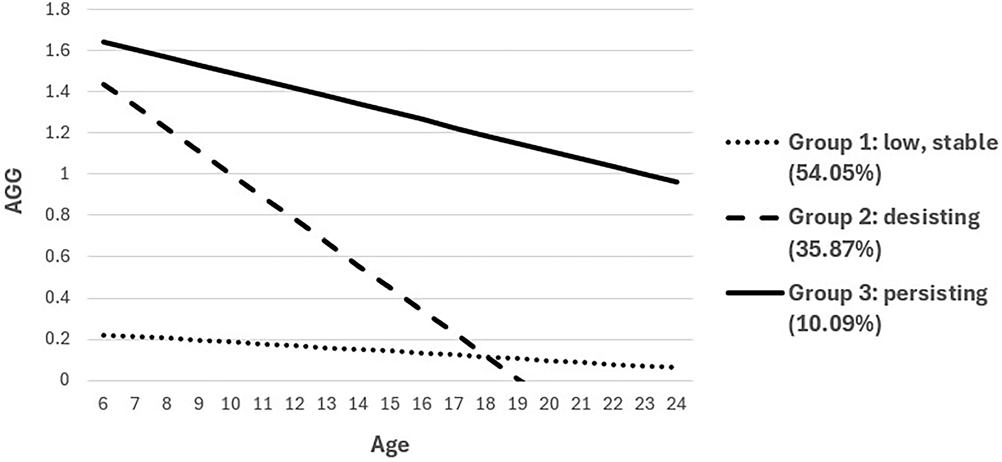
Unconditional three-group model of AGG trajectories (person-centered).

Initial analyses compared the respective fits of a one-class model through to a six-class model to test the hypothesis that there would be either three or four distinct AGG trajectories. Model fit improved as the number of classes increased, as shown in Table 3, but the four through six-class models yielded log-likelihood values that were not replicated and were therefore considered unstable solutions. The three-class solution was thus selected as the optimal model. As shown in Figure 2, the three-class model comprised a low, stable trajectory representing the majority (54.05%) of the sample, as well as two trajectories characterized by high levels of AGG at baseline. One of the latter trajectories (35.87% of the sample) followed a desisting pattern with age, whereas the other group (10.09%) continued to aggress at high levels.

**Table 3.** LCGA fit statistics and predictors of group membership

	Unconditional LCGA model fit statistics				
	−2lnL	AIC	BIC	SABIC	Smallest group (% of sample)
# of groups					
1	6359.74	6369.74	6397.87	6381.99	–
2	5772.97	5788.97	5833.99	5808.57	18.71
3	5569.89	5591.89	5653.78	5618.84	10.09
4	Not replicated	–	–	–	–
5	Not replicated	–	–	–	–
6	Not replicated	–	–	–	–
**Conditional LCGA parameter estimates from 3-group model**					
	Persisting v. low/stable				
	Estimate	S.E.		*p*-value	
**ADI**	**.307**	**.141**		**.029**	
**Income**	**−.412**	**.117**		**< .001**	
**Conflict**	**1.268**	**.144**		**< .001**	
Nurturance	.227	.128		.076	
**Sex**	**.619**	**.254**		**.015**	
Race	.142	.316		.653	
	Persisting v. desisting				
	Estimate	S.E.		*p*-value	
ADI	.182	.173		.292	
**Income**	**−.399**	**.144**		**.005**	
**Conflict**	**.314**	**.157**		**.045**	
Nurturance	.147	.154		.339	
Sex	.132	.307		.668	
Race	−.027	.387		.944	
	Desisting v. low/stable				
	Estimate	S.E.		*p*-value	
ADI	.125	.104		.229	
Income	−.012	.110		.910	
**Conflict**	**.955**	**.110**		**< .001**	
Nurturance	.080	.092		.385	
**Sex**	**.488**	**.178**		**.006**	
Race	.169	.258		.513	

Bold font indicates *p* < .05.

#### Conditional model

We subsequently examined predictors of group membership via a series of pairwise comparisons using the R3STEP command. Male sex, higher ADI, lower income, and higher conflict predicted membership in the persisting group relative to the low, stable group. Income and conflict also distinguished between desisting and persisting youth. Namely, youth with high levels of AGG in childhood who resided in more affluent households, as well as those exposed to less conflict, were more likely to desist from AGG over the course of adolescence. See Table 3 for conditional model results and Table 4 for trajectory group characteristics based on most likely class membership. (For simplicity, trajectory group characteristics are reported at each wave, but models were run with age as the metric of time).

**Table 4.** Characteristics of AGG trajectory groups

	Class 1: low, stable	Class 2: desisting	Class 3: persisting
*N*	1109	736	207
% male	46.2	57.0	59.7
Means (SD) [Range]			
ADI	55.65 (22.24) [2-99]	58.15 (23.16) [2 – 99]	62.68 (22.12) [5 – 99]
Income	8.42 (2.60) [1 – 10]	8.24 (2.65) [1 – 10]	7.31 (3.01) [1-10]
Conflict	19.21 (4.09) [12 – 34.5]	22.11 (4.51) [12.5 – 39]	23.37 (4.95) [13 – 37]
Nurturance	41.79 (3.47) [27 – 48]	41.11 (3.57) [22 – 48]	40.76 (3.56) [32 – 48]
AGG T1	.28 (.47) [0 – 4]	2.65 (1.37) [0 – 8]	4.76 (1.98) [0 – 8]
AGG T2	.23 (.59) [0 – 5]	.51 (.84) [0 – 6]	2.89 (1.59) [0 – 8]
AGG T3	.21 (.64) [0 – 7]	.40 (.75) [0 – 4]	3.21 (1.98) [1 – 8]
AGG T1 (log-transformed)	.19 (.32) [0 – 1.61]	1.23 (.36) [0 – 2.20]	1.67 (.43) [0 – 2.20]
AGG T2 (log-transformed)	.14 (.32) [0 – 1.79]	.30 (.44) [0 –1.95]	1.28 (.42) [0 – 2.20]
AGG T3 (log-transformed)	.12 (.32) [0 – 2.08]	.24 (.40) [0 – 1.61]	1.33 (.46) [.69 – 2.20]

*Note.* Trajectory group characteristics are based on participants’ most likely class membership.

### Joint interpretation

Variable-centered and person-centered analyses both indicated a decline in AGG with age. Moreover, male sex, parent-child conflict, low household income, and neighborhood disadvantage all predicted elevated AGG at baseline, evidenced by their prediction of the intercept (variable-centered) and their distinction between the normative group and the elevated, persistent group (person-centered). However, variable-centered and person-centered results differed with respect to predictors of the rate of change. In the former, only sex and parent-child conflict predicted the slope, and results were not in the expected direction (i.e., male sex and greater conflict predicted *faster* decline with age). Such findings likely reflect a statistical artifact, as males and those with greater conflict evidenced higher baseline levels of AGG and thus had more room to decline over time. In sensitivity analyses that modeled the intercept as a predictor of the slope, greater conflict no longer predicted a more rapid decline in AGG; male sex remained a significant predictor of decline, but with an attenuated effect size. The intercept significantly predicted the slope, supporting our interpretation that the counter-intuitive results from the main analyses were indeed artifacts. Notably, the predictors of the intercept remained the same. Results from the growth curve thus indicated that differences in rate of change were largely unrelated to any of the predictors under study. In the person-centered analyses, by contrast, lower levels of conflict as well as familial affluence predicted desistance from AGG among youth demonstrating elevated engagement at baseline. As the desisting and persisting trajectory groups differed primarily by slope, such findings implicate parent-child conflict and socioeconomic disadvantage as predictors of the slope variance. There was thus some overlap between the factors predicting baseline engagement and rate of change, as higher parent-child conflict and lower household income predicted greater AGG at baseline, and the reverse (i.e., lower conflict and higher income) predicted desistance. Nevertheless, male sex and higher ADI significantly predicted greater baseline AGG in the growth curve, but female sex and lower ADI did not predict membership in the desisting trajectory relative to the persisting one. Based on our joint interpretation, the predictors of baseline AGG and desistance only partially overlap. Should we have relied solely on the results from the growth curve, we would have concluded that two of the factors predicting greater engagement at baseline (i.e., male sex and higher parent-child conflict) also predicted desistance, an unlikely prospect. Such findings point to limitations of variable-centered approaches in understanding heterogeneity in rates of change, particularly when examining desistance.

## Discussion

Our study aimed to elucidate the predictors of desistance from AGG across middle childhood and adolescence by jointly interpreting findings from two distinct statistical modeling approaches. Consistent with our hypotheses, the modal pattern was one of declining AGG with age. That is, variable-centered analyses revealed a mean-level decline in AGG across the study period, and all three trajectories identified via person-centered modeling followed either stable or decreasing patterns. These findings are consistent with a large body of literature indicating that engagement in AGG typically begins to decline by school entry and continues to do so throughout adolescence (e.g., Broidy et al., 2003; Ehrenreich et al., 2014), facilitated by age-related improvements in emotion regulation and behavioral inhibition (Burt, 2012). The number and shape of the trajectory groups we identified were also largely consistent with findings from studies spanning middle childhood and adolescence, with a modal trajectory characterized by a group low on AGG and two higher-risk trajectories with differing degrees of desistance (e.g., Becht et al., 2016; Maughan et al., 2000).

Although findings were largely consistent across variable and person-centered analyses, each approach provided unique information about the development of AGG, particularly in relation to associations with the predictors. The variable-centered analyses revealed that baseline levels of AGG differed as a function of sex, mother-child conflict, familial socioeconomic status, and neighborhood deprivation. However, only sex and mother-child conflict were observed to predict the slope, and with small effect sizes. Based on the variable-centered findings, one might conclude that youth largely followed similar patterns of declining AGG with age. The person-centered analyses, however, painted a different picture. The trajectories differed not only by baseline engagement but also in their developmental patterns, with one group accounting for most of the age-related decline observed across the full sample.

Perhaps most noteworthy among the person-centered findings is that the majority of participants with elevated AGG at baseline had desisted by early adulthood. This finding raises a question that person-centered approaches are uniquely positioned to answer, namely, *which specific factors predict recovery from AGG*? In contrast to the variable-centered findings, the factors predicting patterns of change in the mixture models did so in a way that was consistent with theory. Specifically, socioeconomic advantage, as well as parent-child relationships characterized by little conflict, predicted desistance among youth demonstrating elevated AGG at baseline. Not only do these findings suggest potential targets for intervention within families, but they also point to the utility of person-centered approaches to the study of AGG (and other forms of psychopathology) even within dimensional data. Because variable-centered analyses examine development across entire samples, they are not positioned to extract subgroups that differ widely in their patterns of change, or to identify specific environmental factors predicting such different patterns. Moreover, although person-centered models of antisocial behavior subdivide a continuous trait into distinct categories (e.g., Walters & Ruscio, 2013), the present findings indicate that such approaches can yield information about developmental patterns in continuously distributed data that variable-centered techniques cannot.

We next discuss each of the salient predictors in more detail. As hypothesized, socioeconomic advantage in both the familial and neighborhood contexts predicted less engagement in AGG. In the growth curve model, youth from wealthier households and advantaged Census block groups demonstrated lower levels of AGG at baseline than their peers from impoverished backgrounds. Likewise, in the person-centered analyses, high income and low ADI both predicted stable/low AGG trajectories relative to persistence, and high income predicted desistance among those youth with elevated AGG at baseline. These findings are consistent with theoretical work pointing to numerous environmental contexts (e.g., family, school, neighborhood) that impact child development (Bronfenbrenner, 1988). They also extend prior, cross-sectional research implicating socioeconomic disadvantage as a multi-faceted construct, with non-interchangeable effects from the family environment and the broader neighborhood context (Carroll et al., 2024; Kupersmidt et al., 1995). Youth who reside in homes and neighborhoods equipped with adequate resources are thus more likely to exhibit low levels of AGG. Moreover, even if they do develop AGG during childhood, those from wealthier homes are far more likely to desist than their peers from impoverished backgrounds.

Aspects of the parent-child relationship also predicted trajectories of AGG. Specifically, low levels of mother-child conflict predicted low AGG at baseline in the variable-centered analyses, consistent with prior studies of parent-child conflict and youth behavior problems (El-Sheikh & Elmore-Staton, 2004; Xu et al., 2021). Likewise, in the person-centered analyses, low levels of conflict predicted both low and desisting AGG trajectories relative to persistent AGG. Notably, conflict was one of only two variables that predicted the slope of the latent growth curve, and results were inconsistent with those of the person-centered analyses, such that the former found higher levels of conflict to predict a *faster* decline, and the latter found lower levels of conflict to predict desistance. The unexpected findings in the variable-centered analyses likely reflect regression to the mean among participants demonstrating high levels of AGG at baseline. That is, participants reporting higher levels of conflict also demonstrated greater baseline AGG and thus had more room to decline with age. Indeed, when explicitly modeling the intercept as a predictor of the slope in follow-up analyses, parent-child conflict no longer predicted the slope, suggesting that differences between participants in rate of change were not related to any of the familial or socioeconomic predictors we examined. The prospect of certain variable-centered findings reflecting regression to the mean artifacts is consistent with prior simulation results. Marsh & Hau (2002), for example, simulated test score data in which schools differed by average achievement at baseline but followed identical growth trajectories. Nevertheless, estimates from multilevel growth curve analyses indicated that schools with lower initial averages demonstrated greater growth in scores over time. An analogous pattern was observed in the present data, namely that participants with higher initial AGG scores, including those with elevated parent-child conflict, appeared to decline more with age.

By contrast, when we examined predictors of trajectory group membership, results for conflict were in the expected direction, with lower conflict predicting both consistently low and desisting trajectories. Because participants in the desisting group had greater conflict than those in the normative group, who had little room to decline with age, higher conflict was associated with more rapidly declining AGG in analyses that collapsed across the full sample. Although reflective of the high levels of conflict among participants with elevated AGG at baseline, variable-centered findings thus provided a somewhat misleading picture of the factors predicting the slope variance. Joint interpretation of the variable-centered and person-centered results demonstrates that greater conflict predicts elevated AGG at baseline *and* continued engagement in AGG across adolescence.

Contrary to our hypotheses, however, nurturance did not serve as a protective factor. Prior work has yielded mixed results regarding associations between positive parenting and child behavior problems. For instance, Yingling and Bell (2016) found that parental involvement with their children was unrelated to both baseline level of AGG and rate of change when accounting for other familial characteristics, including socioeconomic status. In addition, Waller and colleagues (2018) examined differences within twin pairs in parental warmth at Wave 1 in the present data (i.e., the TBED-C) and found them to predict twin differences in callous-unemotional traits, but not AGG. In other words, positive parenting may be more relevant to some types of externalizing than to others. The role of positive parenting as a protective factor may also involve more nuance than was able to be detected here. Wang (2019), for example, found high levels of warmth from one parent to serve as a buffer against adolescent AGG in the presence of harsh treatment by the other parent. We focused here on maternal nurturance and were thus unable to examine maternal and paternal differences in parenting as potential moderators. Other studies have found parental nurturance to have a small association with AGG, but at different ages for boys and girls (Arım et al., 2011) or only in conjunction with early pubertal timing in girls (Mrug et al., 2008). In short, additional work is needed to understand how parental nurturance impacts youth behavioral trajectories, including when in development it is most impactful and for whom.

As hypothesized, males demonstrated higher levels of AGG at baseline and were more often assigned to the persistent trajectory group relative to the normative group. Sex also predicted the slope of the growth curve, with males exhibiting a somewhat faster decline in AGG with age, which may again reflect regression to the mean (although males did not exhibit lower levels than females on average prior to age 17; see Figure S1). Notably, however, sex did not distinguish between desisting and persisting trajectory group membership, as both groups were predominantly male. Such findings are fully consistent with the broader literature, which has found AGG to be highly sex-specific throughout early development, with some studies identifying up to 15-fold higher rates of AGG for males relative to females (e.g., Berkout et al., 2011).

### Limitations

Our results should be interpreted in light of several limitations. First, participants were predominantly white. Although our sample is socioeconomically diverse and representative of the racial/ethnic makeup of the State of Michigan, future studies should examine whether our findings generalize to youth from more diverse racial/ethnic backgrounds as well. Next, youth in our study were twins. Prior work has found twins to be comparable to singletons on most traits (Christensen & McGue, 2020), including aggression and other externalizing behavior problems (Pulkkinen et al., 2003). We thus do not expect our findings to differ substantially in singleton samples, but future studies should seek to confirm this.

Next, participants were assessed on up to three occasions across a broad developmental period. The greatest strength of such an approach is its ability to provide insight into behavioral changes throughout nearly all of early development. Nevertheless, it was not possible to model non-linear changes in AGG. This may be particularly salient when modeling the development of AGG for adolescents in under-resourced contexts, whose engagement often fluctuates more than that of their wealthier peers (e.g., Ehrenreich et al., 2014; Karriker-Jaffe et al., 2008). Our study was not positioned to detect such fluctuations, although our finding that familial deprivation predicted persistent AGG is consistent with prior findings that youth exposed to disadvantage are less likely to follow a steadily declining AGG trajectory (Carroll et al., 2023). Future studies of AGG should employ both variable-centered and person-centered approaches while leveraging a different assessment schedule than in the present study (i.e., multiple waves of data collection across relatively brief intervals).

Next, our measure assessed the frequency, not the severity, of AGG, and thus did not detect the age-related increases in severity suggested in prior research (e.g., Odgers et al., 2008). In addition, items assessed behaviors of relatively low severity (e.g., hot temper, mean) rather than violent crime, which may explain why we did not identify a “late-onset” or “adolescent-increasing” group in the person-centered analyses. Studies identifying groups with onset after childhood typically examined serious acts of violence (e.g., shooting, stabbing) as the outcome and did not assess for the presence of less serious forms of AGG (e.g., Mata & van Dulmen, 2012; Tung & Lee, 2017). In short, our finding that AGG was most frequent at study onset (i.e., between ages 5 and 6) is consistent with the broader AGG literature, which has found that AGG rarely first emerges during adolescence or adulthood (Carroll et al., 2023). Future studies are needed to elucidate associations between familial and neighborhood risk factors and the specific types of aggressive behaviors comprising very high-risk trajectories.

Next, we focused on maternal reports of AGG, as they were available at all waves. Although such an approach eliminates confounding due to differences between participants in the number of available informants (i.e., teacher-report data were available for most, but not all, participants at the first two waves), there are inherent limitations to focusing on a single informant. For example, parents may not be aware of the extent of their children’s AGG, particularly during adolescence, given age-related increases in independence and ability to conceal problem behaviors (Burt, 2012). In addition, parent reports largely reflect behaviors occurring in the home and may not adequately capture AGG in other settings (e.g., school; Achenbach et al., 1987). We thus computed mean scores based on data from all available informants (i.e., mother, teacher, and self-report) and examined them as the outcome in a series of sensitivity analyses. As shown in Tables S7 and S8 and Figure S2, results were generally consistent with those from the main analyses. In the growth curve model, ADI, income, conflict, and sex predicted the intercept, in the expected direction, and higher conflict and male sex predicted a greater age-related decline. In the LCGA, in turn, the three-class solution yielded a “low” AGG group, as well as two groups characterized by higher engagement at baseline. The percentage of the sample belonging to the “low” group was less than that assigned to the analogous group based on maternal reports. Moreover, the group with the greatest age-related decline demonstrated more engagement at baseline than the other two groups, meaning that the two higher-risk groups differed not only by slope but also by intercept. Altogether, these findings indicate that parents reported somewhat less AGG than did other informants, fully consistent with prior research on informant effects (Achenbach et al., 1987). As in the main analyses, higher ADI, lower income, greater parent-child conflict, and male sex predicted greater engagement in AGG. Our primary results thus do not appear to be specific to parent-reported AGG but persist to multi-informant composite reports as well.

A limitation specific to the mixture models was that the average posterior probability was relatively low for the persisting trajectory. In mixture modeling, each participant is assigned to the group for which their posterior probability of membership is highest. Average posterior probabilities are thus a measure of classification accuracy, as higher values indicate greater certainty in class assignment. The average probability for the persisting group was .61, somewhat below the recommended threshold of .70 (Nagin, 2005). This is likely related to the study’s accelerated longitudinal design, with incomplete follow-up at the second and third waves of data collection. Nevertheless, average posterior probabilities for the desisting and low/stable trajectories were .80 and .93, respectively, and missing data analyses indicated that participants did not differ in their Wave 1 AGG as a function of attrition. The three-class solution thus assigned participants to trajectories with a reasonable degree of confidence, despite the planned missingness at later waves. In addition, the mixture models constrained within-class variation in the intercept and slope to zero, consistent with a categorical approach to modeling psychopathology and with the majority of prior longitudinal work on antisocial behavior, which has used this approach (e.g., Nagin et al., 2009; Spano et al., 2010). As a robustness check, we fitted a series of growth mixture models (GMM), with both between and within-class variation in trajectories (Muthén & Muthén, 2000). The three-group model identified one group with consistently low AGG and two other groups that eventually desisted (see Figure S3). Although it was not possible to compare desisting and persisting trajectories, in the conditional analyses, higher ADI, lower income, greater parent-child conflict, and male sex predicted greater engagement in AGG (see Table S9), fully consistent with the results from the LCGA. In short, despite the limitations specific to the mixture models, our findings persisted across person-centered modeling approaches.

Lastly, all predictors of AGG trajectories were modeled as time-invariant based on scores at the middle childhood assessment, when sample size was largest. Although beyond the scope of the present study, changes in neighborhood conditions, neighborhood residence, household income and/or parenting behaviors may elicit changes in AGG. In the case of parenting, the association may be bidirectional (i.e., changes in AGG may evoke specific parenting behaviors, as suggested in prior work (Klahr et al., 2014; Narusyte et al., 2011)). Future studies are needed to examine parallel trajectories of family characteristics and youth AGG, particularly to clarify how parenting and AGG develop in tandem.

### Implications

Despite these limitations, the present study has several important implications. First, our findings underscore the importance of leveraging variable-centered *and* person-centered approaches when studying developmental trajectories, even when the trait in question appears to be dimensional. Variable-centered approaches are integral to understanding the overall, sample-level pattern as well as the predictors of the intercept variance. Given the heterogeneity in trajectories of psychopathology within typical samples, however, the magnitude and direction of change over time cannot be fully captured by a single slope estimate. Nor can we fully understand the predictors of the slope variance when collapsing across the entire sample. Thus, after examining development across the full sample via growth curve modeling, one logical next step is to turn to person-centered techniques to understand developmental patterns in greater nuance. By identifying discrete trajectories that differ not only by baseline level but also by direction/rate of change, person-centered techniques can both model the heterogeneity in participants’ slopes and examine the predictors of this heterogeneity in a more nuanced way than can be done in variable-centered approaches.

In the present study, for example, the conditional variable-centered analyses suggested that socioeconomic status did not explain any variance in participants’ slopes, a finding that is highly inconsistent with prior research (Carroll et al., 2023). Moreover, *low* parent-child conflict was implicated as a risk factor for continued AGG, despite predicting lower levels of AGG at baseline. It was only through the conditional mixture models that we were able to identify socioeconomic advantage and low parent-child conflict as specific predictors of desistance over time, and in a way that was consistent with theory and prior research. In short, joint interpretation of the two approaches yielded a far more complete picture of the development of AGG, as well as a more logical conceptualization of the factors predicting interindividual variation, than either approach provided by itself.

Second, the present study advances our understanding of youth AGG as a developmental phenomenon unfolding within multiple contexts. Individual, familial, and neighborhood characteristics all emerged as important predictors of continuity and change in AGG. In particular, our findings implicate both the family and the neighborhood as critical contexts for the emergence of AGG, given that mother-child conflict, familial poverty, and neighborhood deprivation all contributed significantly to AGG trajectories. Moreover, among those youth with high levels of AGG at baseline, familial characteristics predicted which youth eventually desisted. The distinction between desistance and persistence among those with high levels of a given psychopathology is of great clinical importance, given that all who present for treatment presumably demonstrate elevated symptoms at baseline. Successful courses of treatment would thus be reflected in trajectories of desistance, meaning that factors promoting desistance are important to identify and leverage in targeted interventions.

The present findings specifically point to mother-child conflict as a potential treatment target for reducing youth AGG, consistent with the demonstrated efficacy and effectiveness of parent management training (PMT). PMT, a structured intervention in which parents are taught behavioral strategies to promote their children’s prosocial behavior and discourage conduct problems, has proven effective in reducing aggressive, disruptive, and noncompliant behaviors in children and adolescents (DeGarmo et al., 2004; DeGarmo & Forgatch, 2005; Hagen et al., 2011; Kjøbli et al., 2013). Moreover, studies of treatment mechanisms have found increases in effective parenting (e.g., consistent discipline, positive involvement) to mediate the effects of PMT (DeGarmo et al., 2004; DeGarmo & Forgatch, 2005; Hagen et al., 2011). Our findings also support the use of parent emotion coaching in interventions targeting AGG. These strategies, which foster the development of parents’ active listening skills and understanding of their children’s emotional experiences (Lunkenheimer et al., 2007), have proven effective in bolstering youth emotion regulation and reducing behavioral problems (Lee & Kim, 2019). One goal for future work should be to determine to what extent parent-child conflict specifically serves as a mediator for the effects of PMT and emotion coaching, as well as whether findings are consistent across informants (i.e., mother, father, and child reports of conflict).

Socioeconomic advantage within the home also predicted desistance, consistent with prior work that has found exposure to deprivation to be a potent predictor of externalizing behaviors in particular (Carroll et al., 2024; Snyder et al., 2019). These findings underscore the implications of socioeconomic disadvantage for youth mental health not only at a single timepoint but throughout the early developmental period. In more affluent households, for example, healthy development may be facilitated by greater access to resources, such as recreational facilities, structured activities, and, notably, mental health treatment. Indeed, the markers of desistance identified in the present study may partially reflect treatment effects, as youth from wealthier backgrounds who exhibit high levels of AGG may be better able to access treatment than their counterparts from disadvantaged homes. We do not have any systematic data on participants’ enrollment in therapy and/or treatments received throughout the study period. Future studies should examine access to mental health treatment as a potential mediator of the association between (dis)advantage and desistance from youth AGG. Studies are also needed to determine the extent to which changes in socioeconomic status over time predict trajectories of AGG.

Lastly, future research should employ variable and person-centered techniques to illuminate the effects of (dis)advantage on trajectories of other externalizing behaviors, such as non-aggressive rule-breaking (RB). In contrast to AGG, RB follows a developmental pattern characterized by increasing engagement during adolescence, as well as greater fluctuations and lower rank-order stability (Burt, 2012). One might thus expect to identify trajectories of RB that differ not by degree of desistance but rather by the extent of their *increase* across adolescence. Leveraging variable and person-centered methods would allow us to determine the risk factors that predict large increases in RB over time, which would represent promising targets for treatment efforts.

## Supporting information

10.1017/S0954579425100382.sm001 Carroll et al. supplementary material Carroll et al. supplementary material

## Data Availability

Because of the language in the informed consent document at intake, we cannot post the data publicly, but they can be obtained from the primary author upon reasonable request.

## Supplementary material

The supplementary material for this article can be found at https://doi.org/10.1017/S0954579425100382.
