# Supplementary material for: Understanding desistance from aggression: A joint interpretation of person-centered and variable-centered approaches
Source: Dev Psychopathol. 2025 Aug 11:1–15. Online ahead of print. doi: 10.1017/S0954579425100382 (PMC12752470; doi:10.1017/S0954579425100382)
Supplement: Carroll et al. supplementary material [file S0954579425100382sup001.docx]

**Supplementary Methods**

**Measures**

***AGG***

Teacher reports of AGG (20 items) were obtained at the first two assessment waves via the Achenbach Teacher Report Form (TRF; Achenbach & Rescorla, 2001). Teachers rated the twins’ behaviors during the preceding six months using a three-point scale (0=never to 2=often/mostly true). The teachers of 115 participants were not available for assessment (because the twins were home-schooled or because parental consents to contact the teachers were completed incorrectly, etc.). Our teacher participation rate across both subsamples was 86% at Wave 1 and 50% at Wave 2, with teacher reports available for 1,551 and 453 participants, respectively. In addition, the twins completed the Youth Self-Report (YSR) at Waves 2 and 3, reporting on their own AGG (17 items) during the preceding six months using the three-point scale described above (Achenbach & Rescorla, 2001). Reports were available from 99% of the twins at both Waves 2 and 3.

**Data Analytic Strategy**

***IRT Analyses***

Analyses were conducted in M*plus* 8.4 (Muthén & Muthén, 1998-2019) using the weighted least squares mean- and variance-adjusted (WLSMV) estimator. As the twins were nested within families, analyses accounted for the nonindependence of observations using the CLUSTER command. We made use of an IRT model, which relates an individual’s trait level (θ) to their performance on a series of items while accounting for item characteristics. Given the relatively low frequency of AGG in the sample, and consistent with prior IRT analyses of antisocial behavior in the TBED-C (Burt, Donnellan, Slawinski, & Klump, 2016), items were coded dichotomously, with responses of 1 or 2 on the ASEBA instruments collapsed into a single “present” category (i.e., 0=behavior absent, 1=behavior present). We then applied the two-parameter logistic (2PL) model, which estimates two item characteristics: discrimination (α) and difficulty (β) (Embretson & Reise, 2013). Item discrimination indicates the extent to which endorsement of a given item relates to one’s trait level; the higher the discrimination, the more accurately the item assesses standing on the trait of interest. Item difficulty indicates the trait level needed to have a 50% chance of endorsing the item. A higher difficulty thus indicates that one would need a relatively high standing on the latent trait continuum to receive a 1. Such an item would be diagnostic of high levels of AGG.

Initial models included all AGG items from each available informant (i.e., 18 items from the CBCL and 20 items from the TRF at Wave 1; 18 items from the CBCL, 20 from the TRF, and 17 from the YSR at Wave 2; 18 from the CBCL and 17 from the YSR at Wave 3). Each wave comprised one latent factor. Items were omitted from subsequent models based on their characteristics (i.e., low discrimination, low endorsement rate, or standardized discrimination > 1) as well as theoretical considerations (i.e., whether they assessed emotional dysregulation or verbal AGG rather than physical AGG). Model fit was evaluated with three indices: root mean square error of approximation (RMSEA), Comparative Fit Index (CFI), and Tucker-Lewis Index (TLI). RMSEA values below .06 and CFI and TLI values above .95 are considered to indicate good fit (Hu & Bentler, 1999). For each model, we examined modification indices to identify sources of misfit and added the suggested residual covariances between items to the model (omitting covariances involving mother-reported “disobedient at home”, as these were unable to be computed for the anchor item). AGG mean scores were computed based on participants’ responses to the items retained in the final IRT model.

**Supplementary Results**

**IRT Modeling**

The initial IRT model at each wave included all ASEBA AGG items from all available informants. The second model omitted items assessing emotional dysregulation (e.g., sudden changes in mood) and/or verbal AGG (e.g., argues). The final model excluded these items as well as the item “threatens others”, which had a standardized discrimination value greater than 1 when reported by teachers (likely due to its low endorsement rate). Fit indices are reported in Table S3, and parameter estimates from the first two models are reported in Tables S4 and S5, respectively. The final model at each wave retained eight items from each available informant. Fit was good at Waves 1 and 3, according to the RMSEA and CFI. The TLI indicated some degree of misfit at all waves, as did the CFI at Wave 2. Nevertheless, the RMSEA was well below recommended cutoffs at all waves (Hooper, Coughlan, & Mullen, 2008; Hu & Bentler, 1999; Steiger, 2007), indicating good fit. Discrimination and difficulty parameter estimates from the final model are reported in Table S6. As expected, items assessing behaviors that could be considered illegal (e.g., destroys things belonging to others, physically attacks people) were more difficult to endorse than were those assessing less extreme behaviors (e.g., disobedient at home, hot temper). Teacher-reported items generally discriminated better than did other informants’ and were more difficult to endorse at Wave 1 than parent-reported items, consistent with prior work identifying somewhat higher rates of psychopathology in parent reports relative to teacher reports (Ferdinand et al., 2003). Mean scores were computed based on the items retained in the final model. Scores were multiplied by 24 to correspond to the highest number of items included at any wave (i.e., Wave 2) and then log-transformed to account for positive skew.

**Table S1.** Measures included in Area Deprivation Index.

| ***Measure*** |  |
| --- | --- |
|  | 1. Percent of population aged 25 and older with <9 years of education |
|  | 1. Percent of population aged 25 and older with at least a high school diploma |
|  | 1. Percent of population aged 16 and older in white-collar occupations |
|  | 1. Median family income |
|  | 1. Income disparity (ratio of households with <$10,000 income to households with ≥$50,000 income) |
|  | 1. Median home value |
|  | 1. Median gross rent |
|  | 1. Median monthly mortgage |
|  | 1. Percent of housing units owned by occupiers |
|  | 1. Percent of population aged 16 and older who are unemployed |
|  | 1. Percent of families below poverty level |
|  | 1. Percent of population below 150% of the poverty threshold |
|  | 1. Percent of households with children under age 18 headed by a single parent |
|  | 1. Percent of households without a motor vehicle |
|  | 1. Percent of households without a telephone |
|  | 1. Percent of occupied housing units without complete plumbing |
|  | 1. Percent of households with more than 1 person per room |

*Note.* Participating families’ ADI scores were determined by the level of deprivation in their Census block group based on all indices listed above. For additional details, see Singh (2003) and Kind & Buckingham (2018).

**Table S2.** Youth AGG by age.

| Age | Total *N* | Mean AGG (SD) | Range AGG |
| --- | --- | --- | --- |
| 5-6 | 618 | 1.85 (2.04) | 0-8 |
| 7 | 416 | 1.56 (1.72) | 0-7 |
| 8 | 338 | 1.63 (1.96) | 0-8 |
| 9 | 344 | 1.27 (1.71) | 0-8 |
| 10 | 342 | 1.31 (1.71) | 0-8 |
| 11 | 114 | 1.04 (1.40) | 0-7 |
| 12 | 84 | .71 (1.21) | 0-7 |
| 13 | 96 | .79 (1.47) | 0-8 |
| 14 | 199 | .78 (1.47) | 0-8 |
| 15 | 286 | .59 (1.15) | 0-6 |
| 16 | 230 | .40 (.92) | 0-6 |
| 17 | 216 | .71 (1.33) | 0-8 |
| 18 | 132 | .45 (1.09) | 0-7 |
| 19 | 66 | .20 (.56) | 0-3 |
| 20 | 26 | .12 (.43) | 0-2 |
| 21 | 15 | .00 (.00) | 0-0 |
| 22 | 6 | .17 (.41) | 0-1 |
| 23-24 | 8 | .00 (.00) | 0-0 |

*Note.* AGG sum scores were computed based on maternal reports on eight CBCL items.

**Table S3.** IRT model fit.

|  |  | **RMSEA (90% CI)** | **CFI** | **TLI** |
| --- | --- | --- | --- | --- |
| **Wave 1** |  |  |  |  |
|  | **Model 1** | .035 (.033-.037) | .955 | .921 |
|  | **Model 2** | .045 (.041-.050) | .962 | .933 |
|  | **Model 3** | .052 (.047-.057) | .964 | .925 |
| **Wave 2** |  |  |  |  |
|  | **Model 1** | .028 (.026-.030) | .838 | .828 |
|  | **Model 2** | .030 (.026-.034) | .905 | .893 |
|  | **Model 3** | .032 (.028-.036) | .888 | .872 |
| **Wave 3** |  |  |  |  |
|  | **Model 1** | .035 (.032-.039) | .899 | .888 |
|  | **Model 2** | .037 (.029-.044) | .947 | .934 |
|  | **Model 3** | .037 (.027-.046) | .957 | .942 |

*Note.* Model 1 contained all ASEBA AGG items from each available informant at a given wave. Model 2 contained all items assessing physical AGG. Model 3 was the final model, containing items that assessed physical AGG and had good psychometric properties.

**Table S4.** Standardized IRT parameter estimates from models containing all ASEBA AGG items.

| **Wave 1** | | | | | | |
| --- | --- | --- | --- | --- | --- | --- |
|  | **CBCL** | | **TRF** | | **YSR** | |
|  | *α* | *Β* | *α* | *β* | *α* | *β* |
| Argues | .40 | -.27 | .91 | .74 | - | - |
| Defiant, talks back | - | - | .86 | 1.22 | - | - |
| Cruelty, bullying, or meanness | .50 | 1.08 | .80 | 1.25 | - | - |
| Demands attention | .68 | .37 | .71 | .80 | - | - |
| Destroys own things | .62 | 1.00 | .79 | 1.63 | - | - |
| Destroys things belonging to others | .63 | .94 | .81 | 1.84 | - | - |
| Disobedient at home* | .75 | .00 | - | - | - | - |
| Disobedient at school | .74 | 1.05 | .90 | 1.12 | - | - |
| Many fights | .37 | 1.42 | .77 | 1.72 | - | - |
| Physically attacks people | .45 | 1.06 | .81 | 1.73 | - | - |
| Screams | .48 | .83 | .77 | 1.97 | - | - |
| Explosive and unpredictable | - | - | .87 | 1.63 | - | - |
| Easily frustrated | - | - | .76 | 1.19 | - | - |
| Stubborn, sullen, or irritable | .41 | .20 | .89 | 1.13 | - | - |
| Sudden changes in mood | .36 | .64 | .76 | 1.29 | - | - |
| Sulks | .22 | 1.05 | .67 | 1.20 | - | - |
| Suspicious | .41 | 1.67 | .77 | 1.97 | - | - |
| Teases | .51 | .90 | .69 | 1.52 | - | - |
| Hot temper | .57 | .26 | .84 | 1.53 | - | - |
| Threatens people | .48 | 1.51 | .81 | 2.00 | - | - |
| Unusually loud | .26 | .75 | .67 | 1.39 | - | - |
| **Wave 2** | | | | | | |
|  | **CBCL** | | **TRF** | | **YSR** | |
|  | *α* | *β* | *α* | *β* | *α* | *β* |
| Argues | .52 | -.29 | .74 | .07 | .26 | -.36 |
| Defiant, talks back | - | - | .93 | .18 | - | - |
| Cruelty, bullying, or meanness | .60 | 1.09 | .88 | .33 | .42 | .45 |
| Demands attention | .54 | .43 | .74 | .34 | .23 | .56 |
| Destroys own things | .61 | 1.04 | .64 | 1.13 | .51 | .92 |
| Destroys things belonging to others | .69 | .84 | .73 | 1.14 | .56 | 1.32 |
| Disobedient at home* | .71 | .00 | - | - | .36 | .26 |
| Disobedient at school | .73 | .77 | .88 | .34 | .54 | .83 |
| Many fights | .68 | 1.23 | .87 | .82 | .54 | 1.19 |
| Physically attacks people | .63 | 1.23 | .85 | 1.02 | .53 | 1.33 |
| Screams | .59 | .74 | .84 | 1.22 | .52 | .72 |
| Explosive and unpredictable | - | - | .96 | .62 | - | - |
| Easily frustrated | - | - | .82 | .48 | - | - |
| Stubborn, sullen, or irritable | .45 | .03 | .70 | .29 | .02 | -.18 |
| Sudden changes in mood | .47 | .23 | .80 | .40 | .29 | -.22 |
| Sulks | .48 | .62 | .71 | .59 | - | - |
| Suspicious | .49 | 1.04 | .86 | .87 | .31 | .39 |
| Teases | .45 | .62 | .75 | .53 | .21 | .55 |
| Hot temper | .54 | .34 | .92 | .69 | .42 | .12 |
| Threatens people | .72 | 1.28 | 1.00 | .85 | .55 | 1.18 |
| Unusually loud | .56 | .50 | .82 | .63 | .35 | .03 |
| **Wave 3** | | | | | | |
|  | **CBCL** | | **TRF** | | **YSR** | |
|  | *α* | *Β* | *α* | *β* | *α* | *β* |
| Argues | .80 | -.51 | - | - | .42 | -.36 |
| Defiant, talks back | - | - | - | - | - | - |
| Cruelty, bullying, or meanness | .76 | .78 | - | - | .43 | .50 |
| Demands attention | .72 | .34 | - | - | .12 | .84 |
| Destroys own things | .81 | .83 | - | - | .40 | 1.18 |
| Destroys things belonging to others | .80 | .87 | - | - | .50 | 1.54 |
| Disobedient at home* | .82 | .00 | - | - | .44 | .40 |
| Disobedient at school | .79 | .67 | - | - | .50 | .88 |
| Many fights | .95 | .94 | - | - | .42 | 1.50 |
| Physically attacks people | .85 | .92 | - | - | .26 | 2.04 |
| Screams | .83 | .59 | - | - | .41 | .95 |
| Explosive and unpredictable | - | - | - | - | - | - |
| Easily frustrated | - | - | - | - | - | - |
| Stubborn, sullen, or irritable | .75 | -.27 | - | - | .16 | -.31 |
| Sudden changes in mood | .77 | -.07 | - | - | .31 | -.11 |
| Sulks | .71 | .52 | - | - | - | - |
| Suspicious | .63 | .80 | - | - | .32 | .56 |
| Teases | .54 | .54 | - | - | .22 | .50 |
| Hot temper | .84 | .12 | - | - | .36 | .28 |
| Threatens people | .90 | .91 | - | - | .39 | 1.56 |
| Unusually loud | .59 | .75 | - | - | .25 | .30 |

*The CBCL item “disobedient at home” was used as the anchor item at each wave for model identification purposes.

**Table S5.** Standardized IRT parameter estimates from models containing all ASEBA items assessing physical AGG.

| **Wave 1** | | | | | | |
| --- | --- | --- | --- | --- | --- | --- |
|  | **CBCL** | | **TRF** | | **YSR** | |
|  | *α* | *β* | *Α* | *β* | *α* | *β* |
| Cruelty, bullying, or meanness | .69 | 1.06 | .82 | 1.25 | - | - |
| Destroys own things | .75 | .99 | .67 | 1.65 | - | - |
| Destroys things belonging to others | .84 | .92 | .75 | 1.84 | - | - |
| Disobedient at home* | .75 | .00 | - | - | - | - |
| Disobedient at school | .73 | 1.05 | .91 | 1.12 | - | - |
| Many fights | .62 | 1.39 | .82 | 1.71 | - | - |
| Physically attacks people | .64 | 1.04 | .86 | 1.72 | - | - |
| Explosive and unpredictable | - | - | .81 | 1.64 | - | - |
| Hot temper | .72 | .24 | .77 | 1.54 | - | - |
| Threatens people | .69 | 1.48 | .87 | 1.99 | - | - |
| **Wave 2** | | | | | | |
|  | **CBCL** | | **TRF** | | **YSR** | |
|  | *α* | *Β* | *α* | *β* | *α* | *β* |
| Cruelty, bullying, or meanness | .55 | 1.17 | .79 | .45 | .40 | .49 |
| Destroys own things | .64 | 1.02 | .63 | 1.16 | .52 | .92 |
| Destroys things belonging to others | .72 | .83 | .72 | 1.16 | .68 | 1.20 |
| Disobedient at home* | .72 | .00 | - | - | .35 | .29 |
| Disobedient at school | .80 | .71 | .86 | .39 | .59 | .78 |
| Many fights | .71 | 1.21 | .90 | .80 | .61 | 1.13 |
| Physically attacks people | .65 | 1.22 | .87 | 1.01 | .51 | 1.36 |
| Explosive and unpredictable | - | - | .95 | .66 | - | - |
| Hot temper | .57 | .32 | .94 | .70 | .42 | .12 |
| Threatens people | .73 | 1.28 | 1.01 | .86 | .49 | 1.25 |
| **Wave 3** | | | | | | |
|  | **CBCL** | | **TRF** | | **YSR** | |
|  | *α* | *β* | *α* | *β* | *α* | *Β* |
| Cruelty, bullying, or meanness | .79 | .75 | - | - | .46 | .45 |
| Destroys own things | .85 | .77 | - | - | .41 | 1.15 |
| Destroys things belonging to others | .83 | .82 | - | - | .54 | 1.49 |
| Disobedient at home* | .81 | .00 | - | - | .40 | .44 |
| Disobedient at school | .84 | .61 | - | - | .51 | .87 |
| Many fights | .92 | .96 | - | - | .49 | 1.41 |
| Physically attacks people | .86 | .90 | - | - | .24 | 2.05 |
| Explosive and unpredictable | - | - | - | - | - | - |
| Hot temper | .79 | .17 | - | - | .40 | .23 |
| Threatens people | .92 | .87 | - | - | .43 | 1.51 |

*The CBCL item “disobedient at home” was used as the anchor item at each wave for model identification purposes.

**Table S6.** Standardized IRT parameter estimates from final model.

| **Wave 1** | | | | | | |
| --- | --- | --- | --- | --- | --- | --- |
|  | **CBCL** | | **TRF** | | **YSR** | |
|  | *α* | *β* | *α* | *β* | *α* | *β* |
| Cruelty, bullying, or meanness | .56 | 1.08 | .81 | 1.25 | - | - |
| Destroys own things | .73 | .99 | .70 | 1.65 | - | - |
| Destroys things belonging to others | .77 | .93 | .77 | 1.84 | - | - |
| Disobedient at home* | .77 | .00 | - | - | - | - |
| Disobedient at school | .76 | 1.05 | .90 | 1.13 | - | - |
| Many fights | .64 | 1.39 | .81 | 1.72 | - | - |
| Physically attacks people | .56 | 1.05 | .85 | 1.73 | - | - |
| Explosive and unpredictable | - | - | .81 | 1.64 | - | - |
| Hot temper | .69 | .25 | .79 | 1.54 | - | - |
| **Wave 2** | | | | | | |
|  | **CBCL** | | **TRF** | | **YSR** | |
|  | *α* | *β* | *α* | *β* | *α* | *Β* |
| Cruelty, bullying, or meanness | .52 | 1.22 | .79 | .49 | .40 | .50 |
| Destroys own things | .64 | 1.04 | .62 | 1.19 | .52 | .94 |
| Destroys things belonging to others | .72 | .86 | .73 | 1.19 | .54 | 1.37 |
| Disobedient at home* | .75 | .00 | - | - | .33 | .32 |
| Disobedient at school | .82 | .72 | .84 | .44 | .53 | .87 |
| Many fights | .73 | 1.21 | .91 | .82 | .61 | 1.16 |
| Physically attacks people | .67 | 1.22 | .88 | 1.04 | .52 | 1.37 |
| Explosive and unpredictable | - | - | .96 | .68 | - | - |
| Hot temper | .57 | .34 | .93 | .74 | .41 | .14 |
| **Wave 3** | | | | | | |
|  | **CBCL** | | **TRF** | | **YSR** | |
|  | *α* | *β* | *α* | *Β* | *α* | *Β* |
| Cruelty, bullying, or meanness | .72 | .83 | - | - | .43 | .50 |
| Destroys own things | .86 | .78 | - | - | .41 | 1.17 |
| Destroys things belonging to others | .83 | .83 | - | - | .53 | 1.51 |
| Disobedient at home* | .82 | .00 | - | - | .45 | .40 |
| Disobedient at school | .85 | .61 | - | - | .49 | .90 |
| Many fights | .93 | .97 | - | - | .49 | 1.42 |
| Physically attacks people | .87 | .90 | - | - | .26 | 2.04 |
| Explosive and unpredictable | - | - | - | - | - | - |
| Hot temper | .78 | .20 | - | - | .41 | .22 |

*The CBCL item “disobedient at home” was used as the anchor item at each wave for model identification purposes.

**Table S7.** LGM fit statistics and parameter estimates for multi-informant mean AGG scores.

| **Unconditional LGM model fit statistics** | | | | | |
| --- | --- | --- | --- | --- | --- |
| Model | -2lnL | *χ^2^* (df) | AIC | BIC | SABIC |
| **Linear growth** | **8798.09** | **-** | **8814.09** | **8859.13** | **8833.71** |
| Means model | 8934.84 | 136.75† (3) | 8944.84 | 8972.99 | 8957.10 |
|  | | | | | |
| **Conditional LGM parameter estimates** | | | | | |
|  | Parameter | Estimate | S.E. | p-value |  |
| **Intercept** | **Mean** | **.986** | **.077** | **<.001** |  |
|  | **Variance** | **.249** | **.066** | **<.001** |  |
|  | **ADI** | **.081** | **.033** | **.014** |  |
|  | **Income** | **-.107** | **.034** | **.002** |  |
|  | **Conflict** | **.399** | **.027** | **<.001** |  |
|  | Nurturance | .027 | .027 | .332 |  |
|  | **Sex** | **.286** | **.055** | **<.001** |  |
|  | Race | .015 | .081 | .853 |  |
| **Slope** | **Mean** | **-.022** | **.010** | **.023** |  |
|  | **Variance** | **.002** | **.001** | **.003** |  |
|  | ADI | -.003 | .004 | .441 |  |
|  | Income | .001 | .004 | .787 |  |
|  | **Conflict** | **-.024** | **.004** | **<.001** |  |
|  | Nurturance | -.003 | .004 | .400 |  |
|  | **Sex** | **-.015** | **.007** | **.039** |  |
|  | Race | -.005 | .010 | .598 |  |
|  | Intercept with slope | -.011 | .007 | .104 |  |
|  | **Wave 1 Residual variance** | **.517** | **.043** | **<.001** |  |
|  | **Wave 2 Residual variance** | **.273** | **.022** | **<.001** |  |
|  | **Wave 3 Residual variance** | **.296** | **.031** | **<.001** |  |

Bold font indicates *p*<.05. †Significant change in chi-square at *p*<.05.

**Table S8.** LCGA fit statistics and predictors of group membership for multi-informant mean AGG scores.

|  | |  | **Unconditional LCGA model fit statistics** | | | |
| --- | --- | --- | --- | --- | --- | --- |
|  |  | -2lnL | AIC | BIC | SABIC | Smallest group  (% of sample) |
| # of groups |  |  |  |  |  |  |
| 1 |  | 9092.44 | 9102.44 | 9130.59 | 9114.70 | - |
| 2 |  | 8687.58 | 8703.59 | 8748.63 | 8723.21 | 46.92 |
| 3 |  | 8293.69 | 8315.69 | 8377.62 | 8342.68 | 23.41 |
| 4 |  | 7797.83 | 7825.83 | 7904.65 | 7860.17 | 12.05 |
| 5 |  | Not replicated | - | - | - | - |
| 6 |  | Not replicated | - | - | - | - |
|  | |  |  | | | |
| **Conditional LCGA parameter estimates from 3-group model** | | | | | | |
|  | |  | Moderate/declining v. low/increasing | | | |
|  |  | Estimate | S.E. | *p*-value |  |  |
| ADI |  | .070 | .072 | .333 |  |  |
| Income |  | -.005 | .075 | .950 |  |  |
| **Conflict** |  | **.725** | **.077** | **<.001** |  |  |
| Nurturance |  | .00 | .067 | 1.00 |  |  |
| **Sex** |  | **.284** | **.125** | **.024** |  |  |
| Race |  | .118 | .176 | .501 |  |  |
|  | |  | High/declining v. moderate/declining | | | |
|  |  | Estimate | S.E. | *p*-value |  |  |
| **ADI** |  | **.252** | **.095** | **.008** |  |  |
| **Income** |  | **-.300** | **.081** | **<.001** |  |  |
| **Conflict** |  | **.442** | **.082** | **<.001** |  |  |
| Nurturance |  | .072 | .080 | .368 |  |  |
| **Sex** |  | **.596** | **.164** | **<.001** |  |  |
| Race |  | -.016 | .212 | .940 |  |  |
|  | |  | High/declining v. low/increasing | | | |
|  |  | Estimate | S.E. | *p*-value |  |  |
| **ADI** |  | **.322** | **.099** | **.001** |  |  |
| **Income** |  | **-.305** | **.090** | **.001** |  |  |
| **Conflict** |  | **1.167** | **.098** | **<.001** |  |  |
| Nurturance |  | .072 | .083 | .386 |  |  |
| **Sex** |  | **.880** | **.167** | **<.001** |  |  |
| Race |  | .102 | .225 | .650 |  |  |

Bold font indicates *p*<.05.

**Table S9.** Parameter estimates from the conditional growth mixture model for maternal reports of AGG.

| **Conditional GMM parameter estimates from 3-group model** | | | | | | | |
| --- | --- | --- | --- | --- | --- | --- | --- |
|  | |  | Moderate/declining v. low/increasing | | | | |
|  |  | Estimate | | S.E. | *p*-value |  |  |
| ADI |  | .066 | | .074 | .375 |  |  |
| Income |  | .001 | | .076 | .990 |  |  |
| **Conflict** |  | **.704** | | **.078** | **<.001** |  |  |
| Nurturance |  | .002 | | .066 | .974 |  |  |
| Sex |  | .152 | | .125 | .224 |  |  |
| Race |  | .258 | | .180 | .152 |  |  |
|  | |  | High/declining v. moderate/declining | | | | |
|  |  | Estimate | | S.E. | *p*-value |  |  |
| **ADI** |  | **.189** | | **.087** | **.030** |  |  |
| **Income** |  | **-.302** | | **.081** | **<.001** |  |  |
| **Conflict** |  | **.387** | | **.078** | **<.001** |  |  |
| Nurturance |  | .115 | | .079 | .147 |  |  |
| **Sex** |  | **.577** | | **.158** | **<.001** |  |  |
| Race |  | -.184 | | .210 | .381 |  |  |
|  | |  | High/declining v. low/increasing | | | | |
|  |  | Estimate | | S.E. | *p*-value |  |  |
| **ADI** |  | **.254** | | **.089** | **.004** |  |  |
| **Income** |  | **-.301** | | **.087** | **.001** |  |  |
| **Conflict** |  | **1.091** | | **.090** | **<.001** |  |  |
| Nurturance |  | .117 | | .081 | .146 |  |  |
| **Sex** |  | **.730** | | **.158** | **<.001** |  |  |
| Race |  | .074 | | .214 | .729 |  |  |

**Figure S1.** Mean patterns of AGG for males and females from the latent growth curve model (variable-centered), based on maternal reports on the CBCL.

**Figure S2.** Unconditional three-group model from latent class growth analyses of AGG trajectories based on multi-informant mean scores.

**Figure S3.** Unconditional three-group model from growth mixture model analyses of AGG trajectories based on maternal reports.

**References**

Achenbach, T. M., & Rescorla, L. A. (2001). *Manual for the ASEBA school-age forms & profiles: Child behavior checklist for ages 6-18, teacher’s report form, youth self-report: An integrated system of multi-informant assessment*. University of Vermont, research center for children, youth & families.

Burt, S. A., Brent Donnellan, M., Slawinski, B. L., & Klump, K. L. (2016). The phenomenology of non-aggressive antisocial behavior during childhood. *Journal of abnormal child psychology*, *44*, 651-661.

Embretson, S. E., & Reise, S. P. (2013). *Item response theory*. Psychology Press.

Ferdinand, R. F., Hoogerheide, K. N., Van Der Ende, J., Visser, J. H., Koot, H. M., Kasius, M. C., & Verhulst, F. C. (2003). The role of the clinician: three‐year predictive value of parents’, teachers’, and clinicians’ judgment of childhood psychopathology. *Journal of Child Psychology and Psychiatry*, *44*(6), 867-876.

Hooper, D., Coughlan, J., & Mullen, M. (2008). Evaluating model fit: a synthesis of the structural equation modelling literature. In *7th European Conference on research methodology for business and management studies* (Vol. 2008, pp. 195-200).

Hu, L. T., & Bentler, P. M. (1999). Cutoff criteria for fit indexes in covariance structure analysis: Conventional criteria versus new alternatives. *Structural equation modeling: a multidisciplinary journal*, *6*(1), 1-55.

Kind, A. J., & Buckingham, W. R. (2018). Making neighborhood-disadvantage metrics accessible—the neighborhood atlas. *The New England journal of medicine*, *378*(26), 2456.

Muthén, L. K., and B. O. Muthén. 1998-2019. Mplus User’s Guide. Eighth Edition. Los Angeles, CA: Muthén & Muthén.

Singh, G. K. (2003). Area deprivation and widening inequalities in US mortality, 1969–1998. *American journal of public health*, *93*(7), 1137-1143.

Steiger, J. H. (2007). Understanding the limitations of global fit assessment in structural equation modeling. *Personality and Individual differences*, *42*(5), 893-898.
